# Dataset on the antecedents of career adaptability among undergraduate students in Malaysia

**DOI:** 10.1016/j.dib.2020.106211

**Published:** 2020-08-21

**Authors:** Olawole Fawehinmi, Khulida Kirana Yahya, Mohd Yusoff Yusliza, Zikri Muhammad

**Affiliations:** a Faculty of Business, Economics and Social Development, Universiti Malaysia Terengganu, Kuala Nerus, Terengganu, Malaysia; b School of Business Management, Universiti Utara Malaysia, Sintok, Kedah, Malaysia

**Keywords:** Career adaptability, Self-esteem, Pro-active personality, Social support, Final year undergraduate students, Malaysia

## Abstract

This data was obtained from a cross-sectional survey of 188 final year Bachelor of Business Administration (BBA) students in a Malaysian Public University. The survey was self-administered to final year BBA students undergoing seminar course prior to the commencement of their industrial training in the coming semester. A regression analysis was conducted in order to assess the link between students’ self-esteem, pro-active personality, and social support with their career adaptability using the Statistical Package of the Social Sciences (SPSS) 20. Specifically, this data article provides data about the participants’ demographic characteristics, as well as the mean, standard deviation and reliability of the measured constructs. It is believed by the authors that the dataset will guide policy makers on the choice of predictor(s) that could boost the level of students' career adaptability, most especially during the school to work transition, in this current volatile labour market.

**Specifications Table**

**Table:** 

**Subject**	**Applied Psychology**
**Specific subject area**	Career Adaptability
**Type of data**	Table
**How data were acquired**	Survey of final year undergraduate students. Instruments: Statistical Package of the Social Sciences (SPSS) 20
**Data format**	Raw Filtered Analyzed
**Parameters for data collection**	To be included in the sample, the participants had to be identified as Bachelor of Business Administration (BBA) final year undergraduate students taking seminar course before commencing industrial training in the coming semester.
**Description of data collection**	The self-administered survey was distributed conveniently to the students in the lecture hall, at the end of the seminar course class.
**Data source location**	Institution: Universiti Utara Malaysia (UUM) City/Town/Region: Sintok, Kedah Country: Malaysia 6°27′16.79" N 100°30′11.99" E samples/data: Bachelor of Business Administration (BBA) Final year undergraduate students.
**Data accessibility**	Data are included in this article.
**Related research article**	O. O. Fawehinmi, K. K. Yahya, Investigating the linkage between proactive personality and social support on career adaptability amidst undergraduate students. Journal of Business and Social Review in Emerging Economies, 4(1), 2018, 81–92. https://doi.org/10.26710/jbsee.v4i1.370 [1]

**Value of the Data**

• This data provides a significant information on capturing the career adaptability of students through assessing their level of social support, self-esteem and proactive personality.
• This data is important because policy makers can determine new recruits that possess career competencies based on their level of career adaptability.
• Policy makers in higher educational institutions can benefit from these data by increasing students’ social support, pro-active personality and self-esteem in order to heighten their career adaptability.
• This data can be used to assess the mediation effect of self-esteem between social support, pro-active personality and career adaptability.
• These data can be reused by assessing the influence of social structure on the students’ social supports, pro-active personality and self-esteem towards their career adaptability.

## 1 Data Description

The data is portrayed in five tables. Table 1 exhibits the demographics of participants portraying participants’ demographic characteristics, such as gender and age. It also displays the participants’ social structure, such as parents' or guardians’ income and educational level. Next, Table 2 presents the mean, standard deviation and reliability of measured constructs. Table 3 displays the model summary such as the coefficient of the determination (R^2^) of the model. Table 4 demonstrates the ANOVA result of the model. Lastly, Table 5 shows that coefficient of the model, such as the Beta and standard error of the measured constructs.

**Table 1:** Demographics of participants (N = 188)

Variables	Category	Frequency	Percentage (%)
Gender	Male	44	23.4
	Female	144	76.6
	Total	188	100
Age	21	0	0
	22	64	34.0
	23	107	56.9
	24	13	6.9
	Others	4	2.1
	Total	188	100
Marital status	Single	185	98.4
	Married	3	1.6
	Divorced	0	0
	Total	188	100
Ethnicity	Malay	123	65.4
	Chinese	55	29.3
	Indian	6	3.2
	Others	4	2.1
	Total	188	100
No. of Siblings	0	4	2.1
	1	22	11.7
	2	30	16.0
	3	42	22.3
	4	31	16.5
	5	21	11.2
	6 or more	38	20.2
	Total	188	100
Most Influence on Education and Career Plan.	Parents	149	79.3
	Siblings	15	8.0
	Friend	19	10.1
	Others	5	2.7
	Total	188	100
Highest Education level of Parents/Guardian	Primary school	9	4.8
	Secondary school	26	13.8
	SPM	27	14.4
	STPM (A levels)	26	13.8
	Certificate	3	1.6
	Diploma	11	5.9
	Bachelor degree	79	42.0
	Master degree	3	1.6
	MD/PhD or other advanced degree	3	1.6
	Others	1	0.5
	Total	188	100
Income of Parents/Guardian (Monthly)	Less than RM2,000	84	44.7
	RM2,001–RM4,000	66	35.1
	RM4,001–RM6,000	19	10.1
	RM6,001–RM8,000	10	5.3
	RM8,001–RM10,000	4	2.1
	RM10,001 and above	5	2.7
	Total	188	100
Source of Tuition fees	Parents	13	6.9
	Family member	1	0.5
	Loans (Other sources)	5	2.7
	Scholarship	14	7.4
	Self-sponsored	5	2.7
	PTPTN (Tertiary education student loan)	150	79.8
	Total	188	100
First to attend University in the family.	Yes	90	47.9
	No	98	52.1
	Total	188	100

**Table 2:** Mean, Standard deviation and Reliability of measured Constructs

	Career Adaptability	Self-esteem	Pro-active personality	Social Support
Mean	4.1722	3.3904	3.8569	4.1587
Std. Deviation	.37907	.44351	.43755	.52910
Cronbach's alpha	0.911	0.690	0.790	0.866

**Table 3:** Model summary

Model	R	R Square	Adjusted R Square	Std. Error of the Estimate	Change Statistics				
					R Square Change	F Change	df1	df2	Sig. F Change
1	.669^a^	.447	.438	.28416	.447	49.590	3	184	.000

a. Predictors: (Constant), support = Social support; sesteem = Self-esteem; pperson = Proactive personality

**Table 4:** ANOVA^a^

Model		Sum of Squares	df	Mean Square	F	Sig.
1	Regression	12.013	3	4.004	49.590	.000^b^
	Residual	14.858	184	.081		
	Total	26.871	187			

a Dependent Variable: cadapt = Career adaptability

b Predictors: (Constant), ssupport = Social support; sesteem = Self-esteem; pperson= Proactive personality

**Table 5:** Coefficient^a^

		Unstandardized Coefficients		Standardized Coefficients		
		B	Std. Error	Beta	t	Sig.
1	(Constant)	1.713	.245		6.989	.000
	sesteem	-.014	.047	-.016	-.295	.769
	pperson	.391	.053	.452	7.392	.000
	ssupport	.240	.044	.335	5.431	.000

a Dependent Variable: cadapt = Career adaptability. sesteem = Self-esteem; pperson = Proactive personality; ssupport = Social support

The Questionnaire and raw data are attached to the article as supplemental files. The data initiated descriptive cross-sectional design and surveys were distributed to the BBA final year students based on convenience sampling technique.

## 2 Experimental Design, Materials and Methods

The research was quantitative in nature adopting the survey methodology. Non-probability, convenience sampling was utilised because of the difficulty in obtaining the number of students in the university record. The Questionnaire design was based on past research and adaptations were made where necessary. 257 questionnaires were distributed but 188 respondents were the returned, completely filled questionnaires.

Career adaptability items was developed by [2] with Cronbach's alpha of 0.96 and it includes 24 items which are very useful to measure career adaptabilities of individuals in terms of four psychosocial career adaptability resources which are concern, control, curiosity and confidence. Respondents were required to provide responses based on their level of career adaptability through the division of the questions according to the four constructs of career adaptability through these 24 questions measurement using a five-point Likert scale from 1- indicating strongly disagree to 5 - indicating strongly agree. This item has been used by several studies [3,4] and obtained Cronbach`s alpha ranging between 0.91 and 0.95.

Self-esteem scale is the 10-item Likert version developed by [5] containing five positive worded items and five reverse worded items with Cronbach`s alpha of 0.90. Respondents were required to provide responses based on their level of self-esteem through the 10 questions measurement using a five-point Likert scale from 1- indicating strongly disagree to 5 - indicating strongly agree. This item has been used by previous studies [6] and achieved a Cronbach's alpha of 0.85.

Proactive personality of respondents was measured by [7] scale measurement with the Cronbach's alpha of 0.87. Respondents were required to provide responses based on their level of proactive personality through these 10 questions measurement using five-point Likert scale from 1- indicating strongly disagree to 5 - indicating strongly agree. This item has been used by previous studies [1] and obtained cronbach's alpha of 0.79.

The Multidimensional Scale of Perceived Social Support (MSPSS) which was developed by [8] with Cronbach's alpha of 0.91. The 12-item scale consists of three 4-item subscales assessing family support, friend support, and significant other support using a five-point Likert scale from 1- indicating strongly disagree to 5 - indicating strongly agree. This item has been used by earlier studies [1], with the Cronbach`s alpha of 0.86.

## 3 Ethical statement

Since this was a non-experimental, voluntary survey, no ethical approval was required. Nevertheless, the permission of participants were requested in filling out the survey which was conducted anonymously.

## Declaration of Competing Interest

The authors declare that there are no known competing financial interests or personal relationships that could have seemed to impact the work reported in this research data.

## Appendix Supplementary materials

Image, application 1
